# Patterns and Drivers of Packaged (Fortified) Maize Flour Purchase in Urban and Peri-Urban Kenya

**DOI:** 10.9745/GHSP-D-24-00240

**Published:** 2026-08-10

**Authors:** Semeni Ngozi, Ayala Wineman, Mywish K. Maredia, David Tschirley, Ian Fisher, Nahian Bin Khaled

**Affiliations:** a Department of Agricultural Economics and Business, University of Dar es Salaam, Dar es Salaam, Tanzania.; b Department of Agricultural, Food, and Resource Economics, Michigan State University, East Lansing, MI, USA.; c Global Child Nutrition Foundation, Seattle, WA, USA.

## Abstract

**Background:**

Large-scale food fortification is a widely applied strategy to address micronutrient deficiencies, and in Kenya, all industrially processed and packaged maize flour is mandated to be fortified with micronutrients. However, policymakers lack information regarding the population’s readiness to procure fortified foods. This study aims to measure the purchase rate of packaged (and presumably fortified) maize flour among households in two Kenyan cities and assess how food environment, household characteristics, and individual shopper perceptions influence consumption of this product.

**Methods:**

We conducted a survey of 1,507 households in urban and peri-urban Kisumu and Nairobi from May to June 2022 to gather information on food purchases, food-shopping behavior, awareness of fortification, and household demographics and socioeconomic status. Between June and August 2022, we conducted a census of food outlets to characterize the households’ home food environment. To analyze the data, we first conducted a descriptive analysis with statistics disaggregated by study-region, household poverty status, and/or status as a consumer of packaged maize flour. We then used a probit regression to determine the differential influence of various factors on the households’ likelihood of purchasing packaged (presumably fortified) maize flour.

**Results:**

Across Kisumu and Nairobi, 67% of households purchased packaged maize flour, with higher rates seen in urban and peri-urban Nairobi (68% and 70%, respectively) and lower rates seen in urban and peri-urban Kisumu (50% and 34%, respectively). While almost 100% of households had some access to packaged maize flour in their home food environment, the density of outlets that sell this product varied from 12/km^2^ in peri-urban Kisumu to 54/km^2^ in urban Nairobi. Moreover, households that purchased packaged maize flour resided in neighborhoods with approximately 11 more outlets/km^2^, on average, than other households. The local price premium charged for packaged maize flour compared to unpackaged maize flour was a negative and statistically significant driver of the purchase decision.

**Conclusions:**

If the goal is to enhance the population’s uptake of fortified maize meal, the government should encourage the private sector to invest more in food outlets that sell packaged maize flour; reduce the retail cost of packaged maize flour; raise awareness of the benefits of fortification (particularly in peri-urban Kisumu); and make relevant technologies available at the scale of smaller mills.

## Abstract (precis)

Two of three residents living in and around two major Kenyan cities—Nairobi and Kisumu—are reached by large-scale fortification of maize flour. Both poor and non-poor consumers benefit, although price is a deterrent in some settings.

## Teaser

**Key Findings:**

- Two of three residents living in and around two major Kenyan cities—Nairobi and Kisumu—are reached by large-scale fortification of maize flour, and both poor and non-poor consumers benefit from Kenya’s fortification mandate.
- One of three people are not being reached by this mass fortification policy. These are people who reside predominantly in peri-urban Kisumu, in areas with a lower density of food retail outlets offering the fortified product, and where packaged maize flour is sold at a relatively higher price than non-packaged maize flour.
- Some shopper characteristics—shoppers younger in age, those who are female, and those who notice signs that encourage healthy eating and value fortification as a food attribute—are strongly associated with decisions to purchase packaged maize flour.

**Key Implications:**

- Governments should target their efforts to promote and expand the availability and affordability of healthy food products, such as packaged (fortified) maize flour, with consideration of geography, age, and gender.
- Government should collaborate with private companies to raise awareness of the benefits of fortification.

## INTRODUCTION

Food insecurity and micronutrient malnutrition (the inadequate intake of essential vitamins and minerals) remain a challenge in many settings worldwide. As of 2021, 2 of 5 people in the world (about 3.1 billion people) cannot afford a healthy diet, an experience that is most common in lower-income countries of Asia and Africa.^1^ Sustainable Development Goal No. 2 (“end hunger, achieve food security and improved nutrition and promote sustainable agriculture”) calls for actions to expand access to nutritious food.^2^ Specifically, target 2.1 focuses on the extent of access to safe and nutritious food, while target 2.2 focuses on the prevalence of malnutrition. Toward this end, large-scale food fortification programs are increasingly being adopted. These programs, which add select micronutrients to commonly consumed foods during processing, aim to improve the nutritional status of the food supply, which is expected to improve target 2.1 and, subsequently, target 2.2.

Thus far, 126 countries have mandatory fortification of salt. This value is 90 for wheat flour, 33 for vegetable oil, and 19 for maize flour.^3^ The introduction of large-scale food fortification programs in countries such as Uganda and Zambia^4^ demonstrate that this approach to delivering much needed micronutrients is feasible even in relatively low-income settings, and programs for iron and folic acid have been found to reduce rates of anemia and neural tube defects in low- and middle-income countries.^5^ Nevertheless, large-scale food fortification programs have had a mixed record in terms of delivery, coverage, and sustainability,^6^ with greater success seen with non-targeted initiatives that cover the whole population, domestic ability to test for fortified product, and low cost of procuring (often imported) pre-mix. Further research is needed in diverse settings to understand whether and how these programs are effective.^7^

Despite recent improvements, malnutrition rates in Kenya remain high, and the Government of Kenya has embraced large-scale food fortification to promote micronutrient adequacy. In 2012, the National Food Security and Nutrition Policy mandated the fortification of maize flour (as well as wheat flour and vegetable oils/fats), and the standards were made explicit in 2015 in CAP 254, Notice No. 157.^8^ Maize flour is widely consumed in Kenya and is the main ingredient in *uji*, a soft porridge commonly eaten for breakfast, and *ugali*, a stiff porridge eaten for lunch and dinner.^9^ This mandate applies to all packaged dry milled maize products, regardless of the size of the processing firm, and the mandated micronutrients for maize flour fortification include iron, zinc, folic acid, and vitamins A, B1, B2, B3, B6, and B12.^4^ Although compliance to national standards in terms of the level of each micronutrient is low,^10^^–^^12^ Kenya’s fortification program remains strategically important, and there is a growing commitment to strengthen enforcement and increase millers’ capacity to comply with fortification standards.^8^

Processed maize flour available in packaged form remains the only channel to deliver the benefits of large-scale food fortification using maize. However, alternatives to processed and packaged maize flour may be preferred, especially among poor consumers who find packaged foods to be prohibitively expensive,^13^ those who prefer whole flour, or those who do not trust processed products. Thus, fortified maize flour would not reach consumers who pay to mill their own maize grain, purchase unpackaged maize flour from local *posho* mills (small maize milling outlets), or choose a different food product entirely.

If packaged maize flour is the vehicle to realize the public health benefits of mass fortification in Kenya, it becomes imperative to track the rate at which it is purchased and consumed and to identify drivers of consumption. Specifically, studies of the determinants of food-related behaviors and decision-making can feed into the design of demand creation strategies and interventions for fortified foods.^14^^,^^15^ The mixed experiences of different large-scale food fortification programs^5^ warrant additional studies in diverse contexts to understand population receptiveness to fortified foods. This can help policymakers characterize packaged food users, acknowledge their understandings and motivations, and develop marketing and other implementation strategies to increase the uptake of fortified products.

Research has shown that consumers’ sociodemographic characteristics can influence their preference for fortified foods.^15^^,^^16^ Particularly in developed countries, women are found to be more interested than men in attaining healthy diets^17^^–^^19^ and demonstrate a higher acceptance and purchase intention toward nutritionally enriched foods.^20^^,^^21^ For example, in Croatia, it was found that women were more interested than men in attaining healthy diets.^19^ In Kenya, it was found that a majority of primary food shoppers who used fortified maize flour were women,^10^ while in Zimbabwe female-headed households were more likely than their male counterparts to consume fortified foods, which, in turn, reduced the proportion of stunted children in their households.^22^

In some settings, older consumers tend to be more eager to adopt disease-preventative eating habits. For example, in Belgium, age was found to be positively associated with acceptance of fortified foods, even if they tasted worse.^21^ Studies have also documented a positive relationship between consumer acceptance of healthy products and higher levels of education^17^ and income.^23^

Considerable evidence also indicates that consumers’ food purchase decisions are influenced by product attributes, including price.^24^^,^^25^ Affordability is especially important in low-income populations,^26^ and in a study of informal settlements in urban Kenya, food prices were regarded as the most salient factors influencing food choices, with food affordability superseding all other considerations, including taste preferences.^27^ Awareness of fortified foods also influences the likelihood of consumption. For example, in Spain, those with prior purchase experience have been found to exhibit a stronger willingness to pay for nutritionally fortified products.^24^ Relatedly, the extent to which consumers are “health-conscious” is relevant for their willingness to purchase fortified foods—a pattern documented in Sri Lanka.^28^

Few studies have examined the introduction of, access to, and use of fortified maize flour in the Kenya context. One study evaluated the extent of adherence to fortification standards among flour mills, finding adherence to be high among large-scale mills (which tend to produce packaged and sealed products) and low among medium- and small-scale mills (which are less likely to be required to fortify their products if they are not packaged and sealed).^11^ Another study applied qualitative methods to assess the policy-enabling environment for large-scale food fortification in Kenya.^29^ The authors concluded that Kenya has a strong record of policy agenda-setting around fortification, has seen moderate success in policy implementation, and remains weak in terms of monitoring and evaluation. Specific challenges in Kenya include threats to the financial sustainability of the program, weak capacity in surveillance and enforcement, and a need to extend support for fortification to medium-and small-scale millers. Nonetheless, Kenya’s fortification program is regarded as being on a positive trajectory.

This article aims to characterize patterns and determine the drivers of packaged (and presumably fortified) maize flour purchase in Kisumu and Nairobi, two large cities in Kenya. It characterizes the extent to which households have access to packaged maize flour and the extent to which they purchase this product—two gaps in policymakers’ knowledge. Our study provides insights into enhancing the effectiveness and reach of Kenya’s national food fortification program, with lessons for other countries as well. Additionally, it offers practical advice for companies involved in producing, marketing, and increasing consumer demand for healthy foods, like packaged (fortified) maize flour, all aimed at improving public health outcomes.

## Research Questions

We explore the following research questions:

1. To what extent do residents of urban and peri-urban Nairobi and Kisumu purchase packaged/sealed (and presumably fortified) maize flour?
2. To what extent do they have access to packaged maize flour, in terms of food outlets selling this product near their homes and the relative prices of packaged and unpackaged maize flour?
3. From where do they purchase packaged maize flour, in terms of food outlets visited and distances traveled?
4. What characteristics of the home food environment, main shopper, and household are associated with the purchase of packaged maize flour?

## METHODOLOGY

### Study Area and Sample Design

The study covers urban and peri-urban areas of Nairobi and Kisumu, with combinations of city and urban status referred to as the 4 “study-regions.” A multi-stage sampling design was followed, with the 4 study-regions identified in the first stage. Then, across all wards (referred to locally as “locations”), we used the 2019 population census to construct an index of neighborhood wealth, which was used to segment the wards into wealth quartiles, or 4 equal groups along the distribution of wealth values, ranging from poorest to wealthiest. (For urban Nairobi, the top wealth quartile was discarded, as these neighborhoods are not comparable with other areas and a low response rate was expected; the remaining wards were again segmented into wealth quartiles.)

In the next stage, we randomly selected 2 wards per wealth quartile. In urban and peri-urban Kisumu, there were 8 and 7 wards, respectively, and all were selected, giving us a total of 31 wards across the 4 study-regions. Thereafter, 2 enumeration areas (EAs) per selected ward were randomly selected. (An EA is a small geographical unit with an average of about 100 households in a village, group of villages, or part of a town or city.^30^)

In the next stage, all households residing within each selected EA were listed, and 23 households per EA were randomly selected to be included in the sample in urban and peri-urban Nairobi and urban Kisumu, while 27 households per EA were selected in peri-urban Kisumu, to reach a target of about 375 households per study-region. Due to some attrition between listing and interview, the actual number of households varies from this target, and the precise number of households sampled in Kisumu and Nairobi were 775 and 732, respectively. As the population size of Kisumu and Nairobi counties is about 300,745 and 1,506,888 households,^30^ per the Yamane formula, this results in a margin of error of 3.7% in each city or 2.6% in the aggregate of these 2 cities.

To delineate each household’s home food environment, we identified the ‘geographic center’ of each EA (i.e., the average x- and y-coordinates of all sampled households in the EA). For the households in each EA, the home food environment is the area within a certain radius of this point. For urban and peri-urban Nairobi, as well as urban Kisumu, this radius is 0.4 kilometers, while it is 0.6 kilometers in peri-urban Kisumu. This radius was determined based on an analysis of household shopping behavior, which showed that over half of the outlets where households shopped were located within a 0.4 (or 0.6) km radius of the geographic center of the EA. Hence, the area of the home food environment is 0.5 km^2^ except in peri-urban Kisumu where it is 1.1 km^2^.

### Data

Data collection took place from May–June 2022, with 1,507 households in the sample. A structured questionnaire was administered to the main shopper in each household, that is, the adult who is primarily responsible for making decisions about household food purchases. The survey collected data on the household’s demographics, socioeconomic status, and food shopping behavior, as well as the main shopper’s values and priorities related to food. The main shopper provided detailed information on food purchases over the previous week, as well as large food purchases that occurred over the previous month that were not already captured. Information was gathered on food items purchased, the outlets from which they were purchased (with geo-coordinates noted), quantities procured, prices paid, distances traveled, whether the product was packaged and sealed, and (where relevant) whether the main shopper noticed whether the product displayed a fortified foods logo.

It should be noted that maize flour purchasing patterns likely vary over the year, particularly for households that produce maize themselves (16% of households). The maize growing seasons in Kenya generally extend from March to September and October to February. The timing of this data collection fell during the non-harvest season. The data can therefore be considered representative of purchase patterns during non-harvest times and should not be interpreted as representative of patterns over the whole year.

Shoppers in Kisumu and Nairobi may not know with certainty whether the maize flour they purchased was fortified. Because Kenyan law mandates that maize flour that is distributed in a packaged and sealed form must be fortified, we assume that maize flour purchased in a packaged and sealed form is fortified and that all other maize flour is not fortified. This seems likely to capture the households’ intentions with regard to fortified maize flour consumption. Nevertheless, it opens the possibility of both type 2 errors (as when fortified maize flour is repackaged for sale in a manner that is not sealed and is therefore not identified as fortified) and type 1 errors (as when a maize flour company makes the flour available in a packaged and sealed form but has not adhered to the law that it be fortified). Seven percent of households purchase maize flour in a manner that could potentially be a type 2 error while the upper bound for type 1 errors is not known.

To characterize the home food environment, a census was conducted of food outlets in each home food environment in June–August 2022. For each food outlet, we collected information on the foods sold and, where appropriate, the products’ fortification status.

### Data Analysis

We first conducted a descriptive analysis with statistics disaggregated by study-region, household poverty status, and/or status as a consumer of packaged maize flour. Key variables used in analysis are summarized in Table 1. We then used a probit regression to determine the differential influence of various factors on the households’ likelihood of purchasing packaged (presumably fortified) maize flour. The probit model is appropriate with a binary dependent variable. This model assumes the underlying latent variable follows a normal distribution and determines the likelihood of an event falling into a category (such as purchasing maize flour that is packaged). The probit model is estimated as:

(1) $$Y_{ijk}=\alpha +\beta \left[HH_{\mathrm{ijk}}\right]+\gamma \left[\mathrm{Home}\;FE_{\mathrm{jk}}\right]+\delta [\mathrm{Regio}n_{k}]+\mu_{ijk}$$

where $Y_{ijk}$ is a binary variable indicating whether household i in home food environment j and region k purchased any packaged maize flour; $HH_{\mathrm{ijk}}$ is a vector of household characteristics; $\mathrm{Home}\;{FE}_{\mathrm{jk}}$ is a vector of characteristics of the home food environment (consistent for all households sampled in each EA); $\mathrm{Regio}n_{k}$ is a vector of region indicators; β, γ, and δ are vectors of coefficients to be estimated; α is a constant term; and $\mu_{ijk}$ is an error term. Equation (1) was first applied to all households, regardless of whether they purchase maize flour, and then to the subset of households that purchase maize flour in any form. A Heckman technique was used to test for bias from having a non-randomly selected sample of maize flour purchasers (finding no bias). Population weights were used in all analyses, and standard errors were clustered at the level of EA. In this study, we consider a *P* value of .10 as statistically significant.

**TABLE 1.** Summary of Key Household Variables

**Variable**	**Description**	**Variable Type**
**Purchase of maize flour**		
Purchased packaged (fortified) maize flour	1= Household purchased maize flour in packaged and sealed form	Dummy
Price paid	Average price paid for maize flour (KES/gram)	Continuous
Distance traveled	Average distance traveled to purchase maize flour (km)	Continuous
**Main shopper characteristics**		
Age of main shopper	Age of the main shopper in the household (years)	Continuous
Secondary school education	1=Main shopper has at least some secondary school education	Dummy
Female	1=Main shopper is female	Dummy
Notice signs	1=Main shopper notices signs that encourage healthy purchases when shopping	Dummy
Notice nutrition information	1=Main shopper notices nutrition information or nutrition labels on packaged foods	Dummy
Notice fortified status	1=Main shopper notices the fortification status of food products before purchase	Dummy
Fortified status: Most important^a^	1=Main shopper reports that fortification is among the most important food attributes	Dummy
Fortified status: Least important^a^	1=Main shopper reports that fortification is among the least important food attributes	Dummy
**Household characteristics**		
Female-headed household	1=Household head is female	Dummy
No. adults	Number of adults in the household	Continuous
No. children	Number of children in the household	Continuous
Poverty likelihood^b^	Likelihood (%) that a household is below US$3.20/day international poverty level	Continuous
More poor	1=Household is above the median poverty likelihood value	Dummy
Less poor	1=Household is below the median poverty likelihood value	Dummy
Farm household	1=Household is involved in farming	Dummy
**Food environment characteristics**		
Packaged (fortified) maize flour in food environment	1= Home food environment has at least one outlet selling packaged (fortified) maize flour	Dummy
No. outlets selling packaged (fortified) maize flour	Number of outlets in the home food environment that offer packaged (fortified) maize flour	Continuous
Density of outlets selling packaged (fortified) maize flour in home food environment	Number of outlets per km^2^ that offer packaged (fortified) maize flour in the home food environment	
Price of packaged (fortified) maize flour	Median purchase price of packaged (fortified) maize flour in the enumeration area (KES/kg)	Continuous
Price premium for packaged (fortified) maize flour	Gap between median price of packaged and unpackaged maize flour in the enumeration area (KES/kg)	Continuous
**Geographic characteristics**		
Nairobi urban	1= Household resides in urban Nairobi	Dummy
Nairobi peri-urban	1= Household resides in peri-urban Nairobi	Dummy
Kisumu urban	1= Household resides in urban Kisumu	Dummy
Kisumu peri-urban	1= Household resides in peri-urban Kisumu	Dummy

^a^ Respondents were presented with a list of 18 food attributes and asked to select their 4 most important and 4 least important attributes.

^b^ This measure of each household’s likelihood of poverty is based on scores given to 10 indicators, including household size, number of habitable rooms and wall material of the dwelling unit, ownership of a television or mobile phone, whether the household contains any member with a disability, and the nature of household members’ work, literacy, and education. The resulting score is converted into probabilities of falling below the US$3.20/day poverty line.^31^ These probabilities are then used to categorize households as having a low or high likelihood of poverty, based on whether they are below or above the median poverty likelihood score.

## DESCRIPTIVE RESULTS

### Rate of Purchase of Packaged Maize Flour

We now address our first research question, “To what extent do residents of urban and peri-urban Nairobi and Kisumu purchase packaged (fortified) maize flour?” Results in Table 2 show that 81% of households in urban Nairobi purchased maize flour (of any type) in the previous month, while 66% in peri-urban Kisumu did so. In peri-urban Kisumu, many people still depend on agricultural activities and may draw on their own production rather than purchasing maize flour from retail outlets. In contrast, households in Nairobi generally access food products from retail outlets, such as a supermarket or *duka* (small traditional retail shop).

**TABLE 2.** Household Purchase of Packaged Maize Flour, Urban and Peri-Urban Nairobi and Kisumu, Kenya, 2022 (N=1,507)

		**Study-Region**				**Poverty Status**	
	**All**	**Nairobi Urban**	**Nairobi Peri-Urban**	**Kisumu Urban**	**Kisumu Peri-Urban**	**More Poor**	**Less Poor**
Household purchased maize flour (%)	80	81	80	70	66	83	77
Household purchased packaged (fortified) maize flour (%)	67	68	70	50	34	66	67
Household purchased packaged (fortified) maize flour, if any maize flour was purchased (%)	84	84	87	72	51	80	87
Number of times packaged (fortified) maize flour was purchased in a month, if any (mean)	4.3	4.2	4.3	5.3	4.7	5.0	3.7
No. of households	1,507	375	357	383	392	817	690

About two-thirds (67%) of all households purchased maize flour that was packaged and sealed (presumably fortified). This value was highest in peri-urban Nairobi (at 70%) and lowest in peri-urban Kisumu (at 34%). Even when focusing on the subset of households that purchased any maize flour, the share that purchased packaged maize flour was 87% in peri-urban Nairobi and 51% in peri-urban Kisumu. It is clear that fortified maize flour is overall less common in Kisumu. Among households that purchased any packaged maize flour, they purchased this product 4.3 times per month, on average. Poorer households purchased packaged maize flour more times per month (5.0) than less poor households (3.7).

### Access to Packaged Maize Flour

Our second research question is, “To what extent do people in urban and peri-urban Nairobi and Kisumu have access to packaged (fortified) maize flour?” Table 3 characterizes the availability of packaged maize flour in households’ home food environments. Almost all of the home food environments in urban and peri-urban Nairobi and urban Kisumu contain selling points for this product. However, compared to these 3 study-regions, it is somewhat less common (at 92%) for households in peri-urban Kisumu to have any access to packaged maize flour in their immediate food environments.

**TABLE 3.** Availability of Packaged Maize Flour in the Home Food Environment, Urban and Peri-Urban Nairobi and Kisumu, Kenya, 2022 (N=1,507)

		**Study-Region**				**Poverty Status**	
	**All**	**Nairobi Urban**	**Nairobi Peri-Urban**	**Kisumu Urban**	**Kisumu Peri-Urban**	**More Poor**	**Less Poor**
% of households whose home food environment contains any selling points for packaged (fortified) maize flour	100	100	100	100	92	100	100
Average number of outlets in the households’ home food environment that sell packaged (fortified) maize flour	42	54	29	17	12	42	41
Average number of outlets per km^2^ in home food environment that sell packaged (fortified) maize flour	82	107	58	33	10	84	81

Table 3 also displays the average number of outlets selling packaged maize flour in the households’ home food environments. Recall that the size of the home food environment is 0.5 km^2^, except in peri-urban Kisumu where it is 1.1 km^2^. On average, households in urban Nairobi have 54 outlets within their home food environment that sell packaged maize flour, while this value is 29 in peri-urban Nairobi, 17 in urban Kisumu, and 12 in peri-urban Kisumu. When the density of selling points is presented per km^2^, the relative scarcity in peri-urban Kisumu is even more stark, with just 10 per km^2^—one-third the density observed in urban Kisumu and less than one-tenth the density observed in urban Nairobi.

Another aspect of households’ access to packaged (fortified) foods is the relative prices of packaged and unpackaged products. Table 4 shows the average prices paid per gram among households that purchased maize flour. Contrary to our expectations, packaged maize flour does not generally seem more expensive than non-packaged maize flour on a per-gram basis. The price of packaged maize flour tends to be higher, on average, in Kisumu than Nairobi, and while average prices in Nairobi for packaged flour are similar or slightly lower than unpackaged flour, there is a sizable gap in the prices in Kisumu. Specifically, the average price of packaged maize flour is 11 KES/gram higher than unpackaged flour in urban Kisumu and 19 KES/gram higher in peri-urban Kisumu.

**TABLE 4.** Prices Paid for Packaged and Unpackaged Maize Flour (mean KES/gram), Urban and Peri-Urban Nairobi and Kisumu, Kenya, 2022 (N=1,507)

		**Study-Region**				**Poverty Status**	
**Type of Maize Flour**	**All**	**Nairobi Urban**	**Nairobi Peri-Urban**	**Kisumu Urban**	**Kisumu Peri-Urban**	**More Poor**	**Less Poor**
Packaged (fortified)	71	71	68	79	83	69	72
Unpackaged (unfortified)	72	74	68	68	64	70	74

Note: To produce these values, the quantities purchased and expenditures made for maize flour over the previous month were first summed within each household to generate a within-household average, and the average was then calculated across households.

### Sources of Packaged Food Products

Our third research question is, “From where do households purchase packaged (fortified) maize flour, in terms of types of food outlets visited and distances traveled?” Table 5 presents the share of households (among those that purchased any maize flour) that sourced either packaged or unpackaged maize flour from each outlet type. Among households that purchased unpackaged (presumably unfortified) maize flour, 36% purchased it from a *duka*, 42% from a posho mill, and 11% from a market. Among households that purchased packaged (presumably fortified) maize flour, most (66%) purchased it from a *duka*, while 21% purchased it from a large supermarket. Generally, most households in Nairobi and Kisumu purchase maize flour from informal food outlets, especially the *duka.*

**TABLE 5.** Outlets Where Households Purchase Maize Flour, by Type of Maize Flour, Urban and Peri-Urban Nairobi and Kisumu, Kenya, 2022 (N=1,194)

	**% of Households**	
**Outlet**	**Unpackaged (Unfortified)** **(N=374)**	**Packaged (Fortified)** **(N=820)**
Duka	36	66
Large supermarket	4	21
Small supermarket	1	8
Posho mill	42	1
Wholesale	2	4
Kiosk	4	3
Market	11	1
Other	4	1

Note: Each value indicates the percent of households (among those that purchase a given type of maize flour) that procure the product from a given outlet. Because households typically frequent multiple types of outlets, these values sum to more than 100%.

Table 6 shows the average Euclidean distances traveled to purchase packaged and unpackaged maize flour. (A very similar pattern is seen when Table 6 is produced using self-reported distances traveled.) Households traveled longer distances, on average, to purchase packaged maize flour compared to unpackaged maize flour. Recall from Table 5 that packaged products are more likely to be purchased in formal outlets such as supermarkets; because these are less common, they are less likely to be found in the immediate vicinity of someone’s home. Households in peri-urban Kisumu traveled the longest distances, on average, for the purchase of maize flour, presumably because food outlets in peri-urban Kisumu are more dispersed. Results also indicate that less poor households traveled longer distances, on average, for the purchase of packaged maize flour (0.8 km), whereas poorer households purchased packaged maize flour at an average distance of 0.4 km.

**TABLE 6.** Mean Distances Traveled for Maize Flour Purchases (km), Urban and Peri-Urban Nairobi and Kisumu, Kenya, 2022

		**Study-Region**				**Poverty Status**	
**Type of Maize Flour**	**All**	**Nairobi Urban**	**Nairobi Peri-Urban**	**Kisumu Urban**	**Kisumu Peri-Urban**	**More Poor**	**Less Poor**
Packaged (fortified)	0.5	0.5	0.6	0.7	1.2	0.4	0.8
Unpackaged (unfortified)	0.4	0.2	0.6	0.5	1.0	0.4	0.3

Note: To produce these values, we first calculated the within-household average distance traveled to purchase each product across the different purchases made in the previous month and then calculated an average across households.

### Characteristics of Households Purchasing Packaged Maize Flour

Table 7 presents characteristics of households in Nairobi and Kisumu, disaggregated by whether the household purchased any packaged maize flour. Households that purchased packaged maize flour had a main shopper who was younger than households that did not purchase any packaged maize flour (35 and 37 years, respectively; *P*=.02). Households that purchased packaged maize flour were also more likely than others to have a female main shopper (at 73% and 63%, respectively; *P*=.03). The two household categories did not differ significantly in the number of adult and child household members or the household’s likelihood of being poor.

**TABLE 7.** Characteristics of Households, by Purchasing of Packaged Maize Flour, Urban and Peri-Urban Nairobi and Kisumu, Kenya, 2022

		**Did Household Purchase Packaged (Fortified) Maize Flour?**		
** **	**All**	**No**	**Yes**	
** **	**Mean (SD)**	**Mean (SD)**	**Mean (SD)**	***P* Value** ^b^
**Main shopper characteristics** ^a^				
Age (years)	35.83 (12.11)	37.36 (12.64)	35.06 (11.77)	**.02**
Secondary school education	0.64 (0.48)	0.61 (0.49)	0.65 (0.48)	.33
Female	0.68 (0.47)	0.63 (0.48)	0.71 (0.45)	**.03**
Notice signs	0.56 (0.50)	0.58 (0.49)	0.55 (0.50)	.45
Notice nutrition information	0.46 (0.50)	0.49 (0.50)	0.44 (0.50)	.29
Notice fortification status	0.32 (0.47)	0.37 (0.48)	0.30 (0.46)	**.08**
Fortified status: Most important	0.06 (0.24)	0.06 (0.23)	0.06 (0.24)	.77
Fortified status: Least important	0.28 (0.45)	0.30 (0.46)	0.27 (0.45)	.43
**Household characteristics**				
Female-headed household	0.29 (0.46)	0.30 (0.46)	0.29 (0.45)	.68
No. adults	1.98 (0.96)	2.00 (1.06)	1.97 (0.90)	.79
No. children	1.43 (1.43)	1.39 (1.45)	1.46 (1.42)	.59
Poverty likelihood (%)	34.07 (26.25)	34.15 (26.30)	34.04 (26.24)	.96
Farm household	0.16 (0.37)	0.19 (0.39)	0.14 (0.35)	.12
**Food environment characteristics**				
Density of outlets selling packaged (fortified) maize flour in home food environment (number/km^2^)	82.25 (53.74)	74.83 (56.61)	85.97 (51.87)	**.006**
Price of packaged (fortified) maize flour (KES/kg)	68.92 (5.41)	69.97 (7.38)	68.39 (3.97)	**<.001**
Price premium for packaged (fortified) maize flour (KES/kg)	1.64 (5.92)	2.20 (7.54)	1.35 (4.89)	**.07**
**Geography**				
Nairobi urban	0.55 (0.50)	0.53 (0.50)	0.57 (0.50)	.34
Nairobi peri-urban	0.36 (0.48)	0.33 (0.47)	0.38 (0.49)	.21
Kisumu urban	0.05 (0.22)	0.07 (0.26)	0.04 (0.19)	**<.001**
Kisumu peri-urban	0.03 (0.18)	0.07 (0.25)	0.02 (0.13)	**<.001**
**No. of households**	1,507	687	820	

^a^ In households with more than one shopper, the age, education, gender, indicators of awareness, and food values of the main shopper refer to the shopper who responded to the survey on behalf of the household.

^b^ *P* values are for a *t*-test for the difference in mean values. Significant *P* values are in boldface (*P*<.01, *P*<.05, and *P*<.10).

Households that purchased packaged maize flour were slightly less likely than others to have their own farm (at 14% and 19%, respectively), a difference that is close to statistically significant (*P*=.12). Farm households may be more likely to produce their own maize and bring their home-produced product to a *posho mill* to have it turned into flour.

The survey asked the main shopper to indicate their level of agreement with a statement that “they notice the fortification label/logo on food products while they shop.” As seen in Table 7, among all shoppers, just 32% either “agreed” or “somewhat agreed” that they notice the fortification status of foods before they buy them. Other studies have also found low levels of information-seeking behavior among food shoppers in Nairobi, with few reading nutrition information or looking for the fortification logo,^32^ and few showing awareness of the term “fortification.”^33^

Contrary to our expectations, a lower share (30%) of shoppers in households that purchased packaged (fortified) maize flour reported that they notice these logos, compared to 37% among other households. This loosely suggests that the logo does not play an important role in food purchase decisions. The survey also asked the main shopper to consider a list of 18 food attributes and indicate the 4 most important and 4 least important attributes. Just 6% of shoppers indicated that fortification status was most important, and 28% indicated that this was their least important concern. These values do not differ in a statistically significant way across households that did and did not purchase packaged (fortified) maize flour.

In terms of the home food environments, households that purchased packaged maize flour resided in neighborhoods with a higher density of outlets that sold fortified maize flour, compared to other households (at an average of 86 outlets/km^2^ and 75 outlets/km^2^, respectively; *P*=.01). This suggests that households with greater access to packaged flour tend to purchase more of this product.

The local prevailing prices for packaged and unpackaged maize flour are captured using the median observed purchase price within each EA. As seen in Table 7, households that purchased packaged maize flour faced a lower price for this product, on average, than households that did not purchase packaged maize flour (at an average of 68 KES/kg and 70 KES/kg, respectively; *P*<.001). This is consistent with the expectation that consumers—especially in low- and lower middle-income countries—are sensitive to price when shopping. Households that opted not to purchase packaged maize flour also faced a larger local price premium for the packaged product, and this difference was statistically significant (*P*=.07).

### ECONOMETRIC RESULTS

Our final research question is, “What characteristics of the home food environment, main shopper, and household are associated with the purchase of packaged (fortified) maize flour?” This section presents an econometric analysis of the correlates of packaged maize flour purchase, employing a probit model as in equation (1). In Table 8, column 1 displays the results of an unconditional model, and column 2 displays the results of a model focused only on the subset of households that purchased any maize flour. The two models are then applied separately to households in Nairobi (columns 3 and 4) and Kisumu (columns 5 and 6).

**TABLE 8.** Correlates of the Purchase of Packaged Maize Flour (Probit Models), Urban and Peri-Urban Nairobi and Kisumu, Kenya, 2022 (N=1,507)

	**All**		**Nairobi**		**Kisumu**	
	**(1) Unconditional**	**(2) Conditional**	**(3) Unconditional**	**(4) Conditional**	**(5) Unconditional**	**(6) Conditional**
Age of main shopper (years)	−0.003	−0.001	−0.003	−0.001	−0.003	−0.003
* P* value	**.03**	.18	**.04**	.25	**.04**	**.01**
Secondary school education	0.020	0.039	0.021	0.040	0.054	0.017
* P* value	.64	.18	.65	.21	.23	.58
Female	0.130	0.020	0.140	0.018	−0.029	0.012
* P* value	**.02**	.52	**.01**	.60	.54	.68
Notice signs	−0.023	0.044	−0.025	0.044	−0.009	0.047
* P* value	.61	**.08**	.62	.11	.80	**.04**
Notice nutrition information	−0.015	−0.033	−0.013	−0.037	−0.002	−0.015
* P* value	.71	.18	.77	.16	.97	.66
Notice fortification status	−0.091	−0.021	−0.095	−0.021	−0.021	−0.023
* P* value	**.03**	.34	**.03**	.39	.72	.42
Fortified status: Most important	0.043	−0.042	0.048	−0.044	−0.035	0.119
* P* value	.61	.44	.62	.43	.59	**.06**
Fortified status: Least important	−0.035	−0.050	−0.041	−0.054	0.008	0.012
* P* value	.31	**.06**	.27	**.05**	.86	.70
Female-headed household	−0.052	−0.022	−0.051	−0.021	−0.014	0.027
* P* value	.33	.44	.37	.48	.82	.23
No. adults	−0.012	−0.004	−0.016	−0.005	0.017	0.010
* P* value	.66	.80	.60	.74	.44	.60
No. children	−0.006	−0.007	−0.009	−0.010	0.015	0.011
* P* value	.64	.53	.54	.46	.23	.16
Poverty likelihood (proportion)	0.029	0.006	0.023	0.019	0.074	−0.018
* P* value	.76	.91	.83	.78	.40	.81
Farm household	0.003	−0.001	0.022	0.006	−0.128	−0.037
* P* value	.94	.95	.61	.82	**.001**	.16
Density of outlets selling fortified maize flour (10s/km^2^)	0.004	0.003	0.003	0.004	0.008	−0.004
* P* value	.59	.28	.68	.27	.56	.47
Price of fortified maize flour in EA (KES/kg)	−0.004	0.001	−0.003	0.001	−0.001	0.010
* P* value	.65	.85	.80	.92	.81	**.01**
Price premium for fortified maize flour (KES/kg)	−0.003	−0.008	−0.001	−0.009	−0.009	−0.006
* P* value	.64	**.08**	.91	**.09**	**.05**	**.02**
Purchased maize flour in *duka*		0.244		0.226		0.423
* P* value		**<.001**		**.002**		**<.001**
Purchased maize flour in posho mill		−0.150		−0.156		−0.121
* P* value		**.02**		**.03**		**.03**
Purchased maize flour in market		−0.051		−0.079		0.120
* P* value		.49		.34		.12
Purchased maize flour in large supermarket		0.327		0.306		0.521
* P* value		**<.001**		**<.001**		**<.001**
Purchased maize flour in small supermarket		0.285		0.258		0.609
* P* value		**<.001**		**<.001**		**<.001**
Purchased maize flour in other type of outlet		0.147		0.129		0.303
* P* value		**.007**		**.04**		**<.001**
Nairobi peri-urban	0.030	0.062	0.024	0.061		
* P* value	.60	**.09**	.71	.11		
Kisumu peri-urban	−0.239	0.047			−0.079	−0.015
* P* value	**.005**	.24			.23	.65
Kisumu urban	−0.087	0.036				
* P* value	.32	.38				
No. of households	1,507	1,125	732	578	775	547
Wald χ^2^	138.2	772.3	49.5	1432.5	225.2	2019.1
P > χ^2^	<.001	<.001	<.001	<.001	<.001	<.001
Pseudo-$R^{2}$	0.05	0.40	0.03	0.38	0.08	0.55

Average marginal effects; standard errors clustered at enumeration area; significant *P* values are in boldface (*P*<.01, *P*<.05, and *P*<.10).

The conditional model could yield biased results if purchase of any maize flour (i.e., self-selection into the sample) is non-random. We therefore applied a Heckman probit model in which the first-stage selection model predicts the likelihood of selecting into the sample of maize flour purchasers. Because the selection equation should have at least one variable that is not in the second equation, measures of shelf space in the home food environment allocated to whole grains and refined grains were included in the first stage. The Wald test of independent equations indicated that the outcome is not significantly different from the outcome obtained by fitting the probit and selection models separately ($\lambda^{2}=0.77,\;P=.38$). This implies that the results of conditional models reported in Table 8 are not biased.

Among all households, those with older main shoppers are less likely to purchase packaged maize flour, with an additional year in age decreasing the likelihood by 0.3% (*P*=.03). Holding all else equal, households with female main shoppers are 13% more likely to purchase packaged maize flour (column 1), and this pattern is most evident in Nairobi (column 3).

Results in Table 8 indicate that a main shopper who takes note of food’s fortification status before making a purchase is 9% less likely to purchase packaged (fortified) maize flour for their household. This correlation is statistically significant only in Nairobi (column 3). At the same time, shoppers who cited fortification as a most important food attribute are 12% more likely to purchase packaged (fortified) maize flour only in the conditional model for Kisumu (column 6). Meanwhile, shoppers who cited fortification as a least important food attribute are 5% less likely than others to purchase packaged maize flour only in the conditional model for Nairobi (column 4).

The local price premium observed for packaged maize flour relative to unpackaged flour is a negative and statistically significant determinant of purchase, particularly in Kisumu (columns 5 and 6). Specifically, an increase in the price premium by 10 KES/kg is associated with a 9% lower likelihood of purchasing packaged maize flour in Kisumu (β=−0.009, *P*=.05). This is consistent with expectations that consumers respond to price signals. While price stands out as a strong driver of purchase decisions, the physical availability (i.e., density) of fortified maize meal selling points in the home food environment does not seem to influence the likelihood of purchase, once other factors are held constant. However, this coefficient is sometimes statistically significant when other controls, such as prices, are omitted from the equation.

In the conditional model (columns 2, 4, and 6), we also control for the outlet types at which the household purchased any maize flour. (Recall that this variable is not mutually exclusive, as households can procure maize flour from multiple types of outlets. Thus, all outlet types are included in the model.) Those who procured maize flour from a *posho* mill were significantly less likely to purchase packaged maize flour, compared to those who purchased maize flour only from other types of outlets. In the other direction, households that purchased maize flour in a *duka* or small or large supermarket were more likely to purchase the packaged version.

## DISCUSSION

This study employed descriptive and econometric analysis to characterize the packaged (fortified) maize flour purchasing patterns of consumers in Kisumu and Nairobi. Several interesting themes emerge from this analysis.

Nearly all households in Kisumu and Nairobi have some access to packaged (fortified) maize flour in terms of physical availability. However, the intensity of access varies considerably across neighborhoods, and households that purchased packaged maize meal resided in home food environments with a higher density of outlets selling this product. If the goal is to enhance uptake of fortified maize meal, the government might focus on encouraging the private sector to invest more in food outlets that sell packaged maize flour. This is especially the case in peri-urban Kisumu, where the average resident faces a home food environment with just 10 outlets per km^2^ that offer packaged maize flour. While such an effort would be challenging, it is aligned with the current administration’s emphasis on private sector growth through the “Bottom-Up Economic Transformation Agenda,” which identifies micro, small, and medium enterprises (MSMEs) as one of several core pillars of transformational inclusive growth.^34^

The price premium for packaged (fortified) maize flour was a statistically significant driver of the purchase decision, with households less likely to purchase this product when the prevailing price premium in their neighborhood was higher. Although the price gap between packaged and unpackaged flour was limited in the pooled sample, packaged maize flour was strikingly more expensive than unpackaged maize flour in Kisumu, where households were least likely to purchase packaged maize flour. To our knowledge, medium- and large-scale maize milling companies are far more prevalent in Nairobi, which may imply that relatively higher transport costs are incurred in Kisumu. It follows that policymakers intent on advancing Kenya’s large-scale food fortification program should aim to bring down the retail cost of packaged maize flour, perhaps by subsidizing transport costs, waiving import duties on fortification premix, or assisting millers in financing the purchase of fortification equipment. These steps, while important, are likely to be challenging in the face of Kenya’s fiscal limitations and heavy reliance on development partners to finance large-scale fortification activities.^29^

Our results indicate that people in Kisumu and Nairobi are, in general, not concerned with fortification. Just 6% of main shoppers viewed fortification as a most important food attribute, while 28% viewed it as least important. If the goal is to increase uptake of fortified flour, the government should collaborate with private companies involved in the production and marketing of fortified maize flour to raise awareness. As uptake is lowest in peri-urban Kisumu, marketing efforts should be deliberately inclusive of this region.

We found a somewhat unexpected pattern whereby noticing the fortification status of products is negatively and significantly associated with purchase of packaged (fortified) maize meal. Other studies have found that providing information on food products may backfire if consumers distrust a certain technological process or development.^35^ In the Kenya context, some consumers are reported to harbor suspicions regarding the fortification process, with concerns that the products are being adulterated in the course of fortification. Some even insist on visiting *posho* mills so they can continually monitor what is done to their maize.^29^ It follows that the government should design educational campaigns so that consumers understand the fortification process and come to recognize the benefits. Nonetheless, a recent assessment of Kenya’s large-scale food fortification program found it to be weak in terms of consumer education and awareness.^29^ While this points to an opportunity for improvement, it also suggests that this would likely be a challenge.

Most households in Nairobi and Kisumu purchase maize flour from informal food outlets, especially the *duka*. This may be because informal food outlets tend to offer items on credit to regular customers, which is especially important for households with irregular incomes. Moreover, while supermarkets tend to sell larger packages of fixed sizes, a more flexible arrangement is seen among traditional retailers.^36^ Additionally, *dukas* are more common in the food environment and located at closer proximity to consumers than supermarkets. These two features of the *duka*—proximity and small package size—are especially conducive to low-income Kenyan consumers who mostly purchase food in small quantities at high frequencies (commonly referred to as *kadogo* in the local language or “small” in English).^36^ Notably, less poor households may be more likely to have regular income and storage space for products purchased in bulk, whereas poorer households may make more frequent purchases if they have irregular income and limited storage space. Along similar lines, less poor households are more likely to shop in supermarkets, which are likely to be located far from where they reside,^37^ and they may use a private car or other transport to reach more distant shops. On the other hand, poorer households are more likely to access shops on foot and visit food outlets within their neighborhood.

It is not surprising that it is more common to source packaged maize flour from supermarkets, as these modern food outlets are most likely to sell industrially processed products that are packaged and sealed. Likewise, it is not surprising that the most common source of unpackaged maize flour is a *posho* mill, as most of the processed flours offered by these small mills are sold in bulk or in unsealed packages that are exempt from the national fortification mandate. It is important to note that our data collection reflects the non-harvest season and likely underestimates the importance of *posho* mills, which may assume a more prominent role immediately after harvest, when farm-households are more likely to have maize in storage. Future research may give attention to purchase patterns that vary over the year to more accurately capture the importance of different outlet types, including *posho* mills.

One policy implication of our study is that the government could provide subsidized fortification technologies to *posho* mills, especially in peri-urban areas where many people access their maize flour through such mills. This recommendation is consistent with other authors who argue that the government can improve uptake of fortified maize flour by making relevant technologies available at the scale of smaller mills.^38^ It is also consistent with the current direction of the Kenya National Food Fortification Alliance, which has historically been chaired by large-scale millers but is now chaired by small and medium-scale millers, signifying a shift in priorities.^29^ Future research should consider the financial and sociocultural feasibility of interventions that take place through *posho* mills.

Our econometric results indicate that households with younger heads are more likely to purchase fortified maize flour. This is not consistent with the pattern observed in Belgium, where older consumers were generally more open to fortified foods.^21^ In Kenya, however, households with younger shoppers may have more exposure to nutrition information and therefore be more inclined to purchase packaged (fortified) maize flour. Along these lines, studies have found that younger caretakers have greater access to nutrition information stemming from exposure to social media and television.^39^ The causal link between exposure to nutrition information through various types of media and openness to purchasing fortified maize flour is another area for future research.

Our results additionally indicate that households with female main shoppers are more likely to purchase fortified maize flour. As noted, it is common in many contexts to find that women are more interested than men in healthy diets and more accepting of nutritionally enriched foods,^17^^–^^20^ and women in Kenya have elsewhere been found to play a significant role in the decision to consume fortified maize flour.^32^ This could imply that education and marketing campaigns to raise awareness of fortification would be more effective if targeted toward women.

In this study, we focused on the purchase of maize flour that was packaged and sealed, noting that this is mandated by Kenyan law to be fortified. However, when asked to report whether the sealed packages contained a fortification logo, respondents did not know in 39% of cases, strongly indicating that many shoppers are not attentive to this feature when making their food choices. Future research on this topic could more directly capture shoppers’ intentions related to the purchase of fortified products to explicitly discern what drives their decisions.

While these policy implications are relevant in Kenya, these lessons can likely be extrapolated to similar settings, such as Tanzania, Uganda, and Zambia. Policymakers in various countries aiming to increase the reach of their programs should therefore give attention to variation in the intensity of access to fortified foods; the price premium that may be present for fortified products; the types of food outlets frequented by relatively poor people; and the population’s awareness of and perspectives on fortification. Governments should collaborate with private companies to raise awareness of the benefits of fortification and should target their outreach efforts with consideration of geography, age, and gender.

## CONCLUSION

Micronutrient deficiencies constitute a heavy disease burden that is borne disproportionately by people living in developing countries.^1^ Mass food fortification has been demonstrated to be an effective public health intervention to increase micronutrient intake and improve nutrition status.^5^ To avail these benefits, the government of Kenya has implemented a policy of fortifying all industrially processed maize flour and mandated it to be sold in sealed packages through retail shops. In this article, we document that 2 of 3 residents living in and around 2 major cities—Nairobi and Kisumu—are potentially reached by this policy, and as intended, both poor and non-poor consumers benefit equally from this fortification mandate. In this regard, the mass fortification strategy appears to be somewhat effective.

On the flip side, our results show that 1 of 3 people are not being reached by this policy. These are people who reside predominantly in peri-urban Kisumu, in areas with a lower density of food retail outlets, and where packaged maize flour is sold at a relatively higher price compared to non-packaged maize flour. On a more positive note, we find some shopper characteristics—i.e., shoppers younger in age, those who are female, and those who notice signs that encourage healthy eating and value fortification as an important food attribute—to be strongly associated with decisions to purchase packaged (fortified) maize flour. We hope these positive and negative determinants of the packaged maize flour purchase decision will provide guidance on where and how governments should target their efforts to promote and expand the availability and affordability of healthy food products, such as packaged (fortified) maize flour.

## Notes

**Peer Reviewed**

**First Published Online:** April 16, 2026.
